# Locoregional therapy of renal cancer

**DOI:** 10.1186/1470-7330-14-S1-O20

**Published:** 2014-10-09

**Authors:** Tze Min Wah

**Affiliations:** 1 Department of Diagnostic and Interventional Radiology, Institute of Oncology, St. James' University Hospital, Leeds Teaching Hospitals Trust, Beckett Street, Leeds, LS9 7TF, UK

## 

This lecture aims to provide an overview of renal cell carcinoma (RCC) and insights into this rapidly evolving treatment – image guided thermal ablative (IGA) therapy. RCC is the commonest kidney cancer and the detection of RCC has increased over the past decade. There are over 8000 and 50,000 new cases/year in the UK and USA respectively [1,2]. The increased detection is due to wider usage of radiology (e.g. ultrasound or computed tomography) and this usually results in the detection of smaller RCC with earlier stage disease [3,4]. In addition, this is also related to the rise in incidence in the general population as a result of smoking and obesity [5-7].

The historical classical clinical triad where patients presented with flank pain, abdominal mass and haematuria is now a rarity and nowadays the majority of the incidentally detected RCCs are smaller and at an earlier stage –T1 stage. However, historical data has suggested that 60% of these small incidentally detected tumours will grow gradually over a period of time [8]. Therefore, there remains a clinical risk with adopting a ‘watchful waiting’ approach for younger patients as these tumours may become symptomatic or metastasize [9].

Open radical nephrectomy (RN) was the gold standard treatment for RCC since it was introduced in 1869 and Robson et al had popularized this treatment over the last 50 years [10].Over the past 10 years, laparoscopic RN is becoming the standard of care with better cosmetic results and shorter recovery time [11]. More recently for locally confined (T1 disease) RCC treatment with nephron sparing surgery (NSS) e.g. open/ laparoscopic PN has demonstrated similar oncological durability to that of the gold standard RN [12,13]. RN is now largely considered an overtreatment for T1a (<4cm) RCC because it is associated with greater nephron loss and earlier onset of chronic kidney disease and also associated with increased cardiovascular events after RN [14,15].

Therefore, the current consensus is that RCC at stage T1a (<4 cm), should be treated with minimally invasive techniques in order to preserve renal function. Nephron sparing surgery (NSS) with either laparoscopic/open PN is advised whenever it is deemed technically possible [16]. Although, the NSS has similar recurrence free and long term survival outcomes as those with RN, NSS remains technically challenging and associated with significant morbidity even in expert hands [14,15].

Given the surgical challenges and the quest to preserve renal function, IGA treatment of the smaller RCC with radiofrequency ablation (RFA), cryoablation (CRYO) and microwave (MWA) has evolved rapidly over the last decade. Today, IGA of RCC has proven to be a safe and effective treatment option and good oncological outcome data is emerging for RFA [17-22] and CRYO [23,24]. In the hands of an experienced interventional oncologist, the primary technical success is now approaching >95% for both IGA with RFA and CRYO. As RFA was introduced earlier, the emerging larger RFA series have demonstrated cancer specific survival of 97-100% with a follow up of 61-78 months [17-22].
